# Passive and social screen time in children with autism and in association with obesity

**DOI:** 10.3389/fped.2023.1198033

**Published:** 2023-07-10

**Authors:** Aviva Must, Misha Eliasziw, Heidi Stanish, Carol Curtin, Linda G. Bandini, April Bowling

**Affiliations:** ^1^ Department of Public Health and Community Medicine, Tufts University School of Medicine, Boston, MA, United States; ^2^ Department of Exercise and Health Sciences, Manning College of Nursing and Health Sciences, University of Massachusetts-Boston, Boston, MA, United States; ^3^ E.K. Shriver Center, UMASS Chan Medical School, Worcester, MA, United States; ^4^ Department of Nursing and Health Sciences, Merrimack College, Andover, MA, United States

**Keywords:** autism spectrum disorder, screen time, obesity, gender, electronic media

## Abstract

**Background:**

Screen time has been identified as a risk factor for childhood obesity, but the media landscape has evolved rapidly. Children with autism tend to be heavy users of screens and have an elevated prevalence of obesity. We know little about screen use patterns among children with autism vs. typically developing (TD) peers and in association with obesity.

**Methods:**

Baseline data from 10,842 participants in the Adolescent Brain Cognition Development Study was used to characterize time spent with child-reported passive screen use (television/movies/watching videos), playing video games, and using social media. Duration of screen time by autism status and gender was summarized as mean time per day; obesity was defined using CDC/WHO criteria. A propensity score analysis was used to create a matched dataset for analysis.

**Results:**

Overall, 1.7% of children were was identified as having autism. Significant mean differences were observed by autism status and gender for both passive viewing and playing video games. Compared to TD children, boys with autism spent more time (2.9 vs. 2.3 h, *p* < 0.001) watching TV, movies or videos, as did girls (3.0 vs. 2.0 h, *p* = 0.002). Compared to TD peers, boys with autism reported more video game time (102.7 vs. 77.5 min, *p* = 0.001), as did girls with autism (64.4 vs. 37.9 min, *p* = 0.03); girls with autism also spent more time on social media sites or video chat (45.5 vs. 21.9 min, *p* = 0.04). Overall, obesity prevalence increased with increasing screen time duration, significantly for passive screen time (*p*-value = 0.002) and texting (*p*-value = 0.02). Associations between obesity and screen time duration did not differ by autism status.

**Discussion:**

Children with autism spend more time playing video games and on passive and social screen activities than their TD peers, with some variations by gender. High rates of social media use among girls with autism and multiplayer video game use among both boys and girls with autism may challenge the notion that the high levels of screen time reflect social isolation in the group. Given potential positive aspects of screen time in children with autism movement to focus on content and context is appropriate.

## 1. Introduction

The first decades of the new millennium have been characterized by an explosion in the types of screens, their availability, and the proportion of leisure time that children are spending engaging with them (1). With this marked increase in screen use, concerns about the impact of this growing pastime on aspects of child health have ensued. Early foci included the association of screen time with the development and maintenance of obesity in children and the impact of screen use on children’s social and emotional development (2).

Child obesity is a multifactorial disease with risk factors operating at the level of the individual, family, community, and beyond. At the individual level, the key factors that appear to be causal include dietary intake, physical activity, sedentary behavior, and possibly sleep duration. Certain frequently prescribed medications that induce weight gain represent an additional risk factor (3). With respect to sedentary behavior, screen time has emerged as a major contributor to overall sedentary time. Screen time may operate directly on energy balance, given that it requires relatively low energy expenditure. It may also exert its effect on energy balance indirectly, by supplanting time spent sleeping, especially for youth. Early studies that established screen time as an obesity risk factor relied on time spent watching television or videos. The key mechanisms proposed included displacement of physical activity, snacking while viewing, exposure to food advertisements, and interference with the quality and quantity of sleep. However, the nature of screens has evolved rapidly over the last two decades, with the addition of social screen time (e.g., Facebook, Twitter, Instagram), video games that include active and social/multi-player games, video chatting, and texting. Whether these other types of screen time are associated with obesity is less clear. Historically, the American Academy of Pediatrics (4), Canadian Pediatric Society (5) the German Federal Health Ministry (6), have recommended limiting total recreational daily screen time to 2 h, but did not make any distinctions by screen time source. Likewise, unlike passive consumption of movies and television, many new screen types involve elements of socialization (7), although their associations with social development remain unclear.

Children with autism are more likely to have obesity than their typically developing peers (8, 9). Autism is a neurodevelopmental disability characterized by persistent deficits in social communication and social interaction across contexts. Autism has seen increasing prevalence over the last several decades (10, 11). Current data suggest approximately 2% of the pediatric population has autism with an estimated male-to-female ratio of about 4 to 1 (12). Several studies have documented higher levels of screen time in children with autism compared to their typically developing peers (13). The impairments in behavior, intellectual capacity, social communication, and/or social interactions that characterize autism may make participation in structured and unstructured forms of physical activity more difficult, potentially increasing the amount of time spent in sedentary behaviors. Specifically, restricted, repetitive patterns of behavior, interests, or activities as described in the DSM-5 for autism may explain elevated time with electronic screens (DSM-5) (14, 15). However, more research is needed to understand additional drivers for screen affinity in this population, including social isolation and self-stimulation. Screen use may also be considered an area of particular strength or skill, given the high levels of engagement and preference for screen-based modalities among individuals with autism (16). Additionally, inasmuch as the large majority of children with autism are male, they dominate the samples of children with autism, with the result that girls with autism are underrepresented in clinical and population research. Growing evidence suggests that autism presents differently in girls than in boys, emphasizing the need for robust samples that can support gender-specific investigations.

The Adolescent Brain Cognitive Development (ABCD) Study is a longitudinal multisite study enrolling a large sample of US school children aged 9 and 10 years. Their open science model (17, 18) provides access to this unique data set and the opportunity to explore how screen use types differ between boys and girls with autism and typically developing children, and to assess whether any associations between autism status and obesity differ by screen time. We hypothesized that the magnitude and direction of differences in the duration of screen time by autism status would vary by screen type reported in ABCD, which include passive screen use (TV, movies, videos), video gaming, and social media/video chatting, and texting. We further hypothesized that the pattern of associations of screen time with obesity would be similar in children with autism and typically developing children.

## 2. Methods

### 2.1. Study population

We used baseline data from the ABCD study of children enrolled from 21 study sites between September 2016 and October 2018. Within study sites, consenting parents and their assenting children were recruited largely from public and private schools. The geographic locations that comprise the ABCD research sites are nationally distributed and generally represent the range of demographic and socio-economic diversity in the US. Information about the sample design, recruitment, measures, and compensation is detailed elsewhere (17, 19). The de-identified data set was deemed non-human subjects research by the Tufts University Institutional Review Board.

### 2.2. Screen time

Screen time was measured using the Youth Screen Time Survey (20). Children answered questions about typical hours per day spent on six different screen activities, separately for weekdays and weekend days. The 7-point scale responses (none, <30 min, 30 min, 1 h, 2 h, 3 h, and 4+ hours) were converted to corresponding count measures for the purpose of analyses (0, 15, 30, 60, 120, 180, and 240 min). In calculating screen time, the six screen activities were classified into four categories: passive viewing (watching television, movies, or videos), playing video games, social media (visiting social networking sites, such as Facebook, Instagram and video-chatting); and texting. For each category, daily screen time was calculated from the weighted average of the weekday and weekend screen time activities: [(sum of weekday activity × 5) + (sum of weekend day activity × 2)]/7.

### 2.3. Autism status

We defined autism status (yes/no) based on a single question on the ABCD screening questionnaire completed by parents, “Has your child been diagnosed with autism spectrum disorder?” ABCD Study inclusion criteria required children to be in regular (mainstream) classes at school; we have classified these children as typically developing.

### 2.4. Weight status

Body mass index (BMI) was based on measures of height and weight, which were taken as the average of up to 3 separate measurements. BMI was calculated as weight in kg divided by height in meters squared. Sex and age-specific BMI z-scores were referenced against the Center of Disease Control 2001 (21). BMI z-scores and classified as obese (≥95th percentile) or not (<95th percentile).

### 2.5. Covariates

Race/ethnicity categories were defined as Hispanic, non-Hispanic White, non-Hispanic Black, non-Hispanic Asian, and non-Hispanic other/multi-race. Individual socioeconomic position (SEP) scores were calculated from a weighted combination of four highest household education levels and four household income levels (22). The SEP scores range from 1 to 10, with higher scores corresponding to higher socioeconomic positions.

### 2.6. Statistical analyses

A propensity score analysis was used to create a matched data set for the analyses. The propensity of being a child with autism was estimated using a generalized linear model with generalized estimation equations. The data were modelled using a binomial distribution with a log link, and included child age, gender, and race/ethnicity, as well as household socioeconomic position as covariates. A compound symmetry correlation structure was used to account for the within-site clustering of observations. The propensity score was used to perform a nine-digit match of one child with autism to five typically developing children using a greedy matching algorithm (23). As propensity score matching ensured comparable distributions of characteristics between children with autism and typically developing children, adjustment for covariates in multivariable regression analyses were unnecessary. Means and proportions were used to summarize continuous and categorical variables, respectively.

Generalized linear models using generalized estimation equations were used to derive the estimates and inferences for duration of screen time analyses. As screen time was considered to be count data and was positively-skewed, a Poisson distribution with a log link yielded the best fitting models. A compound symmetry correlation structure was used to account for the within-site clustering of observations. For each category of activity, the model consisted of two fully-crossed factors (gender and autism status). Durations of screen times were summarized as mean hours per day or minutes per day. Ratio of means with corresponding 95% confidence intervals were used to compare duration of screen time between autism status, separately for boys and girls.

For analyzing obesity, generalized linear models using generalized estimation equations were used to derive the estimates and inferences. The data were modelled using a binomial distribution with a log link. A compound symmetry correlation structure was used to account for the within-site clustering of observations. For each category of activity, the model consisted of two fully-crossed factors (three-category screen time duration and autism status). Prevalence ratios with corresponding 95% confidence intervals were used to compare the prevalence of obesity across categories of screen time duration. All statistical analyses were carried out using SAS 9.4 (SAS Institute Inc., Cary, NC), and results with *p*-values < 0.05 deemed statistically significant.

## 3. Results

A total of 10,842 children from the ABCD study were included in the analyses, of which 187 (1.7%) were reported to have been diagnosed with autism. Propensity score matching yielded a data set consisting of 186 children with autism and 930 typically developing children. One child with autism could not be matched and therefore was excluded from further analyses. The resulting demographic characteristics of the participants were well-balanced (Table 1). Mean (standard deviation) age of participants was 10.0 (0.6) years. The sample was predominantly male (85.5%) and White (53.8%), with 15.6% being Hispanic, 13.4% Black, and 1.6% Asian. Parents were well-educated, with 87.6% reporting post-secondary school educational attainment. Reported household income varied widely, with approximately one-third reporting less than $50,000 annual income and one-third reporting annual income that exceeded $100,000. Among the 186 children with autism, 35 (18.8%) met the definition for obesity compared to 134 of 930 (14.4%) typically developing children.

**Table 1:** Demographic characteristics of the propensity score matched sample.

	Autism	Typical
	(*N* = 186)	(*N* = 930)
Age of child, *n* (%)		
9 years old	84 (45.2)	420 (45.2)
10 years old	102 (54.8)	510 (54.8)
Gender of child, *n* (%)		
Boy	159 (85.5)	795 (85.5)
Girl	27 (14.5)	135 (14.5)
Ethnicity/race of child, *n* (%)		
Hispanic	29 (15.6)	145 (15.6)
White, non-Hispanic	100 (53.8)	500 (53.8)
Black, non-Hispanic	25 (13.4)	125 (13.4)
Asian, non-Hispanic	3 (1.6)	15 (1.6)
Other or Multi, non-Hispanic	29 (15.6)	145 (15.6)
Highest household education, *n* (%)		
Grade school	1 (0.5)	7 (0.7)
High school	22 (11.8)	109 (11.7)
College or vocational	57 (30.7)	273 (29.4)
University or postgraduate	106 (57.0)	541 (58.2)
Household income, *n* (%)		
<$50,000	70 (37.6)	326 (35.0)
$50,000 to $74,999	9 (4.8)	78 (8.4)
$75,000 to $99,999	25 (13.5)	131 (14.1)
$100,000 or greater	69 (37.1)	331 (35.6)
Don’t know	5 (2.7)	35 (3.8)
Refused to answer	8 (4.3)	29 (3.1)
Socioeconomic position, *n* (%)		
Low range	23 (12.4)	115 (12.4)
Medium range	50 (26.9)	250 (26.9)
High range	113 (60.7)	565 (60.7)

Overall, children spent an average of 2.4 h per day watching television or movies or videos, 76.3 min playing video games, 17.1 min visiting social networking sites or video chat, and 12.4 min texting.

Significant differences between boys and girls and between children with autism and typically developing children were observed for both passive viewing (watching TV, movies or videos) and playing video games (Table 2). In comparison to typically developing children, boys with autism spent 26% more time (2.9 vs. 2.3 h) and girls with autism spent 50% more time (3.0 vs. 2.0 h) watching TV, movies or videos, while both boys and girls with autism significantly played about 25 more minutes of video games per day than their typically developing peers. The only other statistically significant screen time difference was among girls with autism who spent 108% more time (45.5 vs. 21.9 min) visiting social networking sites or video chatting. Daily duration of texting was low and was similar across groups.

**Table 2:** Comparison of mean daily screen times by type, gender, and autism status.

Type	Gender	Status	Sample size	Mean	Ratio of means	95% CI	*P*-value
Watch TV, movies or videos hours per day	Boy	Autism	159	2.9	1.26	(1.13*–*1.42)	<0.001
		Typical	795	2.3			
	Girl	Autism	27	3.0	1.50	(1.17–2.01)	0.002
		Typical	135	2.0			
Play video games minutes per day	Boy	Autism	159	102.7	1.32	(1.12–1.56)	0.001
		Typical	795	77.5			
	Girl	Autism	27	64.4	1.70	(1.04–2.77)	0.03
		Typical	135	37.9			
Social and video chat minutes per day	Boy	Autism	159	16.9	1.09	(0.67–1.76)	0.74
		Typical	795	15.5			
	Girl	Autism	27	45.5	2.08	(1.01–4.28)	0.04
		Typical	135	21.9			
Texting minutes per day	Boy	Autism	159	11.4	0.91	(0.61–1.36)	0.65
		Typical	795	12.5			
	Girl	Autism	27	14.6	1.06	(0.63–1.77)	0.83
		Typical	135	13.8			

Considering all children (Table 3), the prevalence of obesity increased significantly with increasing duration of passive viewing (*p* = 0.002), playing video games (*p* = 0.05), and texting (*p* = 0.04). A similar pattern of increased obesity occurrence was also observed for visiting social networking sites and/or video chat, but did not reach statistical significance. There were no significant interactions between autism status and screen time duration in relation to obesity (all *p*-values > 0.05); therefore Tables 4, 5 show similar patterns of increased prevalence of obesity with increasing duration of different types of screen time for both children with autism and typically developing peers.

**Table 3:** Prevalence of obesity by type and duration of screen time for all children.

Duration per day	Sample size	Prevalence *n* (%)	Prevalence ratio	95% CI	*P*-value
Watch TV, movies or videos—all children (overall *p*-value = 0.002)					
Less than 2 h	584	61 (11.0)	Reference		
2 to 3.99 h	306	53 (17.5)	1.59	(1.12–2.26)	0.009
4 h or more	226	55 (23.7)	2.15	(1.40–3.31)	<0.001
Play video games—all children (overall *p*-value = 0.05)					
Less than 30 min	320	35 (11.6)	Reference		
30–59 min	253	35 (14.2)	1.23	(0.90–1.67)	0.19
60 min or more	543	99 (18.3)	1.58	(1.09–2.29)	0.01
Social and video chat—all children (overall *p*-value = 0.58)					
0 min	670	96 (14.8)	Reference		
1–44 min	338	53 (16.0)	1.08	(0.80–1.46)	0.61
45 min or more	108	20 (17.8)	1.20	(0.84–1.73)	0.32
Texting—all children (overall *p*-value = 0.04)					
0 min	671	98 (15.2)	Reference		
1–29 min	306	40 (13.3)	0.88	(0.66–1.17)	0.37
30 min or more	139	31 (21.5)	1.42	(1.00–2.01)	0.05

**Table 4:** Prevalence of obesity by type and duration of passive screen time, and autism status.

Duration per day	Sample size	Prevalence *n* (%)	Prevalence ratio	95% CI	*P*-value
Watch TV, movies or videos—children with autism (overall *p*-value = 0.29)					
Less than 2 h	69	10 (14.9)	Reference		
2–3.99 h	59	10 (16.9)	1.13	(0.46–2.76)	0.79
4 h or more	58	15 (25.4)	1.70	(0.83–3.47)	0.14
Watch TV, movies or videos—typically developing (overall *p*-value = 0.007)					
Less than 2 h	515	51 (10.5)	Reference		
2–3.99 h	247	43 (17.6)	1.68	(1.14–2.49)	0.009
4 h or more	168	40 (23.1)	2.21	(1.35–3.63)	0.002
Play video games –children with autism (overall *p*-value = 0.16)					
Less than 30 min	36	3 (9.0)	Reference		
30–59 min	40	6 (14.4)	1.60	(0.43–5.92)	0.48
60 min or more	110	26 (23.5)	2.62	(0.64–10.8)	0.18
Play video games—typically developing (overall *p*-value = 0.11)					
Less than 30 min	284	32 (11.9)	Reference		
30–59 min	213	29 (14.2)	1.19	(0.84–1.70)	0.33
60 min or more	433	73 (16.9)	1.42	(1.02–1.98)	0.04

**Table 5:** Prevalence of obesity by type and duration of social screen time, and autism status.

Duration per day	Sample size	Prevalence *n* (%)	Prevalence ratio	95% CI	*P*-value
Social and video chat—children with autism (overall *p*-value = 0.82)					
0 min	119	21 (17.6)	Reference		
1–44 min	45	9 (20.0)	1.13	(0.49–2.62)	0.77
45 min or more	22	5 (22.8)	1.29	(0.56–2.98)	0.54
Social and video chat—typically developing (overall *p*-value = 0.80)					
0 min	551	75 (14.2)	Reference		
1–44 min	293	44 (15.3)	1.08	(0.77–1.53)	0.65
45 min or more	86	15 (16.6)	1.17	(0.69–1.97)	0.56
Texting—children with autism (overall *p*-value = 0.42)					
0 min	121	20 (16.6)	Reference		
1–29 min	45	10 (22.3)	1.34	(0.78–2.30)	0.29
30 min or more	20	5 (23.9)	1.44	(0.59–3.47)	0.42
Texting—typically developing (overall *p*-value = 0.01)					
0 min	550	78 (14.8)	Reference		
1–29 min	261	30 (11.7)	0.79	(0.55–1.13)	0.19
30 min or more	119	26 (21.1)	1.42	(0.97–2.08)	0.07

## 4. Discussion

We sought to leverage the detailed screen use data in the ABCD Study of 9- and 10-year-olds to understand how screen use types differed between boys and girls with autism and their typically developing peers. Passive screen time, video games, social screen time, and texting time were explored separately. We also assessed whether any associations between screen time and obesity differed by autism status. Compared to typically developing children, we observed that boys and girls with autism reported more passive screen time. At the same time, both boys and girls with autism played more video games than their typically developing peers. Finally, social screen time and texting were far less frequent overall, but social screen time appeared to be elevated among girls with autism compared to their typically developing girls.

The world of screens has evolved substantially since the early published reports of television viewing and obesity in 1980s (24, 25) with the introduction of social media and mobile technologies. These newer forms of electronic media may function differently with respect to obesity risk. Specifically, playing video games is not likely to be accompanied by excess snacking as is the case with passive viewing, in part because play requires continual manual interaction. Further, to the extent that high levels of exposure to food advertising fuel snack consumption, advertising-free viewing options have increased (26). Importantly, playing video games is more physiologically demanding than passive viewing, as reflected in energy expenditure and other physiologic responses (27). Additionally, active video games demand far greater expenditures of calories compared to passive video games, up to four times greater in one study (28). The explosion of social play options for video games and multiplayer video games has also changed the social-emotional dynamics of screen-based gaming, allowing youth to interact with friends, family, and meet new people while playing. This may alter the implications of high video game play time among youth with autism, as it may be less associated with social isolation and more associated with socialization. Engagement with social media, texting, and chatting may be done in shorter time intervals. Finally, users of handheld devices, smart phones, and tablets may not be sitting; as such, these activities do not comprise sedentary behaviors.

### 4.1. Differences by screen time type and by gender

Mixed findings with respect to differences in screen time patterns in children with autism and typically developing children may reflect the aforementioned changes in the contemporary landscape of digital media as well as the broad age ranges in prior studies. In an analysis of the 2011–2012 National Survey of Children’s Health, Montes et al. found no difference between children aged 6–17 with and without autism in total screen, media, or computer/mobile device time or in adherence to the recommendation to limit screen time to less than two hours daily. Interestingly, in a separate report using the same dataset, Gillette et al. reported that children with autism were more likely to be “never users” of electronic devices than children without autism (29). Two systematic reviews also reached conflicting conclusions. A systematic review that covered the years 2005–2016 and included the Montes et al. report, described mixed findings in the 17 studies reviewed that focused on comparisons of children with and without autism (30). It is noteworthy that in several of the studies, the comparison group comprised children with other developmental disabilities. The more recent systematic review covering publications through 2018 was more conclusive, finding that in 14 of 16 studies, children with autism used more screen media than those without autism (29). Our analysis, which focused on a narrower age range of 9- and 10-year-olds found that passive viewing, videogaming, and social media/video chatting were more frequent among children with autism than among typically developing children, with some notable differences by child gender.

Our study is among the first to report screen time patterns separately for boys and girls with and without autism. Because the ratio of males to females with autism is estimated at a little over 4 to 1 (12), most studies do not report estimates for boys and girls separately. Of the 16 studies included in the 2019 systematic review of screen use and autism, none reported gender-specific results (29). An earlier review of 47 studies also reported no findings separately by gender (13). One notable exception is an investigation by Mazurek et al. that studied a sample of 202 youth with autism aged 8 to 18 years and their typically developing siblings (31). With a broad age range and a sample size of 166 boys and 31 girls with autism, they found that compared to their typically developing siblings, television time was significantly higher in girls with autism but not different in boys with autism (31). Compared to their typically developing siblings, youth with autism of both genders also reported higher levels of video game time, but lower levels of social media use (31). We found elevated passive screen time levels for both genders: boys with autism had 26% more passive screen time and girls with autism had 50% more passive screen time than their typically developing peers. Although social screen time was a less common activity for both genders, there appear to be important gender differences. Whereas we saw no difference by autism status in average social screen time levels for boys, twice the level of social screen time was observed among girls with autism compared to typically developing girls.

Our understanding of gender differences in autism is emerging, with reports that girls with autism are better able to mask their social deficits than are boys. A large meta-analysis study of children with autism found that compared to boys, girls evidenced less repetitive and stereotyped behavior after age 6 but were similar in the domains of social behavior and communication (32). In a mixed methods observational study of playground behavior, girls and boys with autism were found to play differently; girls were more likely to situate themselves near social circles, where even if less likely to engage in talking, they maximized the opportunity for social interaction. In contrast, boys with autism tended to be solitary and did not participate in the structured games that engaged typically developing boys (33). The higher levels of social screen time we observed in girls with autism seems consistent with this desire for social interaction. Social screen time may feel safer than in-person social interactions—i.e., the screen may provide a buffer that facilitates social encounters with peers. This may also drive higher rates of video game use among boys and girls with autism vs. their typically developing peers. Anecdotal evidence from participants in our current exergaming intervention with adolescents with autism suggests they are drawn to engage with peers in online video games. More research is needed in this area to better elucidate the types of video games children with autism are engaging in, including the amount and types of social interaction required and/or chosen.

### 4.2. Associations with obesity

Cross-sectional and longitudinal studies have linked screen time, usually captured as television viewing time, to obesity in typically developing children (25, 34). Positive results of a randomized clinical trial suggest that the association is causal (35). A 2018 meta-analysis of 16 studies estimated that, compared to children watching fewer than 2 h daily, children watching two or more hours were 67% more likely to have obesity (36). Less is known about these associations in children with autism. We previously reported elevated relative weight (BMI z-score) in association with weekend, but not weekday, screen time in a convenience sample of 53 children with autism and 58 typically developing children, aged 3 to 11 years (37). In the present study, we did not find evidence that the association between screen time duration and obesity in 9- and 10-year olds differed by autism status. Although not statistically significant, the patterns of estimates were similar in children with autism and their typically developing peers, with evidence of increasing obesity prevalence with increased passive screen time and time playing videogames or video chatting. We previously identified an age-dependent divergence in obesity by autism status in children ages 10 to 17 years in the nationally representative National Survey of Children’s Health 2011–2012 (38). Obesity prevalence at age 10 did not differ by autism status (prevalence ratio = ∼1) but rose steadily to a prevalence ratio of ∼3 at age 17 (37). Given the longitudinal design of the ABCD study, further exploration of these associations as these children go through adolescence is warranted—one might expect differences to emerge over the adolescent developmental period (39, 40).

Beyond concerns that excess screen time will impair academic or social development, compromise physical activity engagement, or contribute to obesity, managing the screen behavior of children with autism can be source of considerable conflict and stress for parents (40). The classic restrictive and repetitive characteristics that define autism are readily reflected in viewing certain videos repeatedly or playing video games in ways that parents deem excessive (31, 41, 42). Youth may play video games late at night instead of sleeping—which itself is often particularly problematic for this population (42). However, the potential adverse impact on child health and family dynamics associated with screen time should be considered in the contexts of its benefits, particularly for children with autism. These children may use screens in many positive and beneficial ways. In addition to the social interaction afforded by social screen time and some video games as discussed above, interventions have been successful using video to model appropriate behavior (43). In our study of leisure time activities in transition-aged individuals with autism, participants favored videogames and other electronic media, reporting these activities were enjoyable and contributed to their feelings of competence and independence (44). In therapeutic settings, preference for passive viewing and video games are effective motivators or positive reinforcers (30). Parents also view screens as a source of wellbeing for their children with autism, providing essential downtime that their children need to decompress after a day at school (30). Viewing television, movies, and videogames can also serve as a positive shared family activity (30, 45).

### 4.3. Limitations and strengths

There are several noteworthy limitations of our study. First, the autism status of participants was based on parent-reported screening questionnaire, which may be subject to misclassifications. Screen time information was child-reported separately for screen time types. This approach is known to result in overestimations because of the difficulty in separating out the different screen activities as well as multi-tasking (e.g., texting while watching television) (46). Furthermore, we lack information regarding the proportion of videogaming that is active or played online with others (and therefore, social). To the extent that any misclassification of autism status or screen time is random, it would make differences more difficult to discern. Second, although BMI is an indirect measure of obesity, it correlates with more direct measures of body fatness. In addition, examining obesity, rather than overweight as the outcome of interest, minimizes misclassification. Third, one of the eligibility criteria for inclusion in the ABCD Study was being enrolled in regular classes. This criterion likely resulted in the exclusion of children with developmental or behavioral characteristics that would preclude their full inclusion in mainstream programming, such as those with little or no functional communication, and/or children with a co-occurring intellectual disability. The prevalence of autism in the sample, at 1.7%, is lower than the current estimated prevalence in the general population of 2.3% (12). Nonetheless, the size of the study produced a sample of 186 children with autism, including 27 girls. This sample size was adequate to support our planned analyses, but does limit generalizability to populations of children with autism who have greater support needs.

Our study also had several strengths. Screen time was reported by the children themselves, and reported in many distinct categories, supporting a comprehensive investigation. Although, as noted above, the reporting task has its challenges, parents are particularly ill-suited to provide this information. Parents are unlikely to know what their children are watching/doing, especially in the late evening hours when children may be playing video games while their parents believe they are sleeping (47). All participants were 9 or 10 years old, which is an important developmental period for obesity risk (48), and the narrow age range enhances interpretability, particularly for our findings in girls. The design of the study, with 21 recruitment sites located throughout the US, generally represents the range of sociodemographic characteristics of the 9- and 10-year school children nationally. Finally, the propensity score analysis created a dataset that resulted in well-balanced demographic characteristics between the two groups of children, thus not requiring the need for multivariable regression analyses to adjust the results for confounding factors as is required in an observational study.

## 5. Conclusions

Overall, we found that passive screen and video game use was higher among both boys and girls with autism relative to their typically developing peers. Our study identified some important gender differences in viewing patterns by screen time type. In particular, we found higher levels of social screen time in girls with autism compared to typically developing girls. We did not find evidence that the modest association of screen time with obesity in the sample of 9- and 10-year-olds differed between children with and without autism. It will be important to assess these associations longitudinally as children move through adolescence.

Highly prescriptive guidelines (e.g., limiting total leisure screen time to <2 h/day) promulgated by medical professional organizations have largely been replaced by a more balanced approach to screen use policies. The American Academy of Pediatrics updated their guidance for parents in 2016 and now counsel parents to work collaboratively with their children to create a family media use plan that moves beyond setting time quotas (49). The Canadian Pediatric Society 2022 guidelines for young children emphasize quality over quantity (50). These recommendations seem particularly appropriate for families with a child with autism who need support as they consider electronic media content, implications for social interaction, and the context in which their child engages, in addition to being attentive to the total time spent.

## Data Availability

Publicly available datasets were analyzed in this study. Data are available on The National Institute of Mental Health Data Archive (NDA) website.
